# Predictors of Abdominal Aortic Aneurysm Shrinkage after Endovascular Repair

**DOI:** 10.3390/jcm11051394

**Published:** 2022-03-03

**Authors:** Rianne E. van Rijswijk, Erik Groot Jebbink, Suzanne Holewijn, Nicky Stoop, Steven M. van Sterkenburg, Michel M. P. J. Reijnen

**Affiliations:** 1 Department of Vascular Surgery, Rijnstate, 6815 AD Arnhem, The Netherlands; e.grootjebbink@utwente.nl (E.G.J.); sholewijn@rijnstate.nl (S.H.); nickystoop@hotmail.com (N.S.); svansterkenburg@rijnstate.nl (S.M.v.S.); mreijnen@rijnstate.nl (M.M.P.J.R.); 2 Multi-Modality Medical Imaging Group, TechMed Centre, University of Twente, 7522 NH Enschede, The Netherlands

**Keywords:** AAA, EVAR, remodeling, shrinkage, regression, prediction

## Abstract

Recent studies demonstrate that patients with a shrinking abdominal aortic aneurysm (AAA), one-year after endovascular repair (EVAR), have better long-term outcomes than patients with a stable AAA. It is not known what factors determine whether an AAA will shrink or not. In this study, a range of parameters was investigated to identify their use in differentiating patients that will develop a shrinking AAA from those with a stable AAA one-year after EVAR. Hundred-seventy-four patients (67 shrinking AAA, 107 stable AAA) who underwent elective, infrarenal EVAR were enrolled between 2011–2018. Long-term survival was significantly better in patients with a shrinking AAA, compared to those with a stable AAA (*p* = 0.038). Larger preoperative maximum AAA diameter was associated with an increased likelihood of developing AAA shrinkage one-year after EVAR—whereas older age and larger preoperative infrarenal β angle were associated with a reduced likelihood of AAA shrinkage. However, this multivariate logistic regression model was only able to correctly identify 66.7% of patients with AAA shrinkage from the total cohort. This is not sufficient for implementation in clinical care, and therefore future research is recommended to dive deeper into AAA anatomy, and explore potential predictors using artificial intelligence and radiomics.

## 1. Introduction

An abdominal aortic aneurysm (AAA) eligible for treatment can be treated with either open surgical repair (OSR) or endovascular repair (EVAR). Because of its minimally invasive nature, EVAR has an early benefit with regard to morbidity and mortality in comparison to OSR [1,2]. However, long-term mortality and rupture risk of EVAR are increased compared to OSR [1,2].

In the case of EVAR, AAA growth is thought to represent treatment failure as it reflects pressure on the aneurysm wall—indicating the aneurysm is still at risk of rupture despite the intervention [3]. Several studies have shown that this growth is mostly related to the presence of endoleaks [4,5,6]. A stable or shrinking aneurysm diameter after EVAR has traditionally been considered a treatment success. However, recent studies indicate that patients with AAA shrinkage, at one-year after EVAR, have significantly better long-term outcomes compared to patients with growing AAA, but also to those with stable AAA [7,8,9,10,11]. These outcomes include fewer reinterventions and late complications, less rupture, and a lower all-cause mortality [7,8,9,10,11]. These observations were independent of the occurrence of endoleaks and reinterventions performed [7]. Therefore, the view is shifting from AAA growth as a predictor of EVAR failure, to AAA shrinkage as a predictor of EVAR success.

However, as these advances are only recent, it is not yet known what causes one patient to develop a stable AAA while others have a shrinking AAA one-year after EVAR. Lalys et al. performed a systematic review and meta-analysis on predictors of AAA shrinkage, but they studied shrinkage at any time during follow-up and not specifically one-year after EVAR [12]. Furthermore, a recent systematic review of anatomical predictors of AAA remodeling showed that strong consistent evidence on this type of predictor is missing [13]. It is important though to identify predictors of AAA shrinkage since they also indirectly predict positive long-term outcomes after EVAR.

In this study, we therefore investigated a broad range of demographic, clinical, and procedural parameters to identify their potential in differentiating patients that will develop a shrinking AAA from patients who will have a stable AAA at one-year after EVAR. This paper shows that age, preoperative AAA diameter, and infrarenal angle are significantly associated with the development of AAA shrinkage. However, the multivariate logistic regression model was only able to correctly identify AAA shrinkage in 66.7% of the total patient group.

## 2. Materials and Methods

### 2.1. Study Design and Patient Population

A single-center, retrospective, observational study was performed on patients that were electively treated for an infrarenal AAA between 1 January 2011, and 31 December 2018, and had similar imaging available preoperatively and during follow-up. The regional Human Research Committee declared that the study was not subject to the Medical Research Involving Human Subjects Act (case number: 2020–6721). Approval for data collection and publication was granted by the institutional review board (study number: 2021–1836).

Patients were included if they underwent elective EVAR for an infrarenal AAA with initial assisted technical success—according to the reporting standards for EVAR [14]. Patients had to be treated with endografts that are still used in current clinical practice—including the Excluder (W.L. Gore and Associates, Flagstaff, AZ, USA), Endurant (Medtronic, Minneapolis, MN, USA), Zenith (Cook, Bloomington, IN, USA), Incraft (CardinalHealth, Dublin, Ireland), AFX (Endologix, Irvine, CA, USA), and Anaconda (Terumo Aortic, Inchinnan, UK) endografts. Follow-up data of at least one-year was required—including AAA imaging. Either computed tomography (CT)-CT or ultrasound (US)-US was used to compare the preoperative and postoperative AAA diameter. Patients with ruptured, inflammatory, symptomatic, juxtarenal, suprarenal, thoracoabdominal, and thoracal aneurysms were excluded, as well as patients treated with fenestrated or branched endografts, iliac branched devices, non-CE marked devices within a trial setting, or chimney procedures. Furthermore, patients with an AAA-related reintervention within one-year after EVAR were excluded, because both the complication and the reintervention might have influenced the state of AAA remodeling at one-year.

### 2.2. Definitions and Data Collection

Preoperative, perioperative, and early postoperative data were retrospectively derived from the electronic health records of the included patients and entered into an SPSS database (Version 22, IBM Corporation, Armonk, NY, USA). Preoperative neck and iliac vascular characteristics were extracted from the radiologist’s reports of the preoperative CT scan. The preoperative infrarenal β angle between the flow axis of the infrarenal neck and the body of the AAA (°) [15] was measured on the preoperative CT scan using IntelliSpace Portal (Version 11.1, Philips, Best, The Netherlands) for all included patients. The maximum AAA diameter (mm) was also measured with this software on the preoperative and 12 (range 6–18) months postoperative CT scan for all included patients. The diameter was measured in the plane perpendicular to the aortic centerline—in the anterior-posterior direction—from the outer wall to the outer wall [16]. For every patient, two additional variables were computed—the Society for Vascular Surgery/American Association for Vascular Surgery (SVS/AAVS) medical comorbidity grading [15] and the St George’s Vascular Institute (SGVI) risk score [17]. The SGVI score is based on the maximum AAA diameter and largest common iliac artery diameter and serves as a predictor of mid-term reinterventions and endograft complications [18].

The included patients were stratified into two groups based on the difference in maximum AAA diameter preoperative and one-year after EVAR. Because of known poor agreement in diameter measurement between CT and US imaging, pre- and postoperative diameters were only compared if they were measured with the same imaging modality [19]. All US assessments were performed by dedicated vascular technicians. The choice of imaging modality was per discretion of the treating surgeon. If patients had both US and CT imaging preoperatively and one-year after EVAR, the diameter difference on CT was used—except if the date of the postoperative CT was ≤9 months or ≥15 months after EVAR and the US date was within 10–14 months after EVAR. Patients with a postoperative diameter reduction of ≥5 mm were assigned to the shrinkage group, and patients with a diameter difference of <5 mm were assigned to the stable group [14].

### 2.3. Statistical Analysis

Demographic, clinical, procedural, and early postoperative characteristics were presented as counts and percentages for categorical variables, and as means ± standard deviations for continuous variables. These characteristics were computed for the total patient group and stratified by AAA remodeling at one-year after EVAR.

Because of the large sample size (>40), the central limit theorem is invoked to use parametric tests to identify significant differences between the shrinkage and stable groups [20]. Categorical variables were compared using the χ^2^-test and continuous variables were compared using unpaired Student’s *t*-test.

To identify predictors of AAA sac shrinkage after EVAR, univariate analysis was performed based on a binary logistic regression model. All variables with a *p*-value < 0.3 were evaluated for inclusion in multivariable logistic regression analysis with the Enter method. The maximum number of variables that could be included in the multivariable logistic regression model was limited to a minimum of 10 patients per event—defined as the smallest number of patients in one of the two groups [21]. Deciding which specific variables to include was based on the *p*-value of univariate analysis, possible collinearity, and expectations of the predictive value. For the resulting variables, a manual stepwise logistic regression analysis was performed—using Enter—by removing the variable with the largest *p*-value per step, until the remaining variables had a significant *p*-value. These variables were then entered in the final multivariable logistic regression analysis with the Enter method.

Freedom from death was analyzed for shrinking and stable AAA using Kaplan-Meier analysis with a corresponding log-rank test. All statistical analyses were performed with IBM SPSS Statistics for Windows, where a *p*-value < 0.05 was considered statistically significant.

## 3. Results

**Figure 1:**
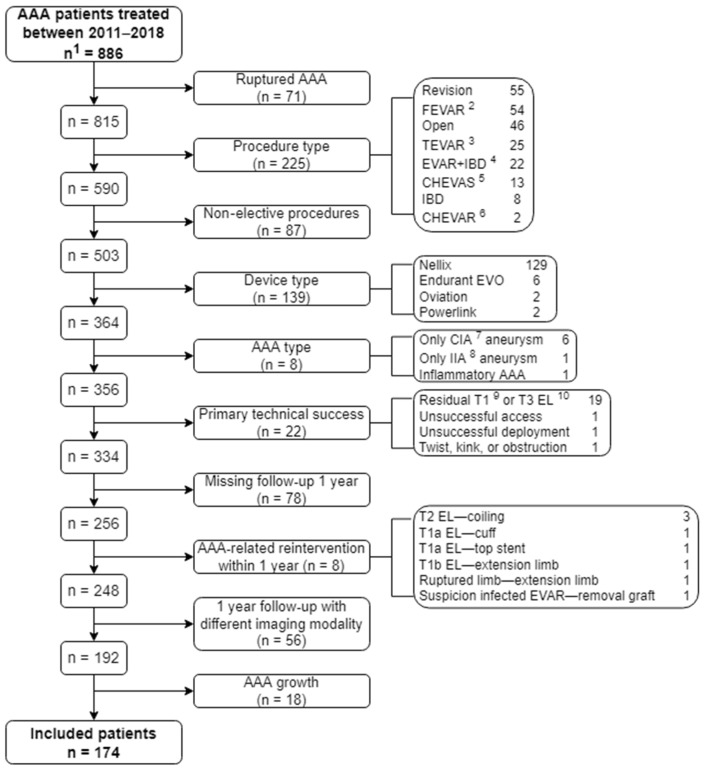
Flowchart of patient selection. ^1^ n = number of patients, ^2^ FEVAR = fenestrated EVAR, ^3^ TEVAR = thoracic EVAR, ^4^ IBD = iliac branched device, ^5^ CHEVAS = chimney endovascular sealing, ^6^ CHEVAR = chimney EVAR, ^7^ CIA = common iliac artery, ^8^ IIA = internal iliac artery, ^9^ T = type, ^10^ EL = endoleak.

A total of 174 patients were enrolled. An overview of the patient selection is given in Figure 1. One-year after EVAR, 67 patients (39%) developed ≥5 mm sac shrinkage, and 107 patients (62%) had a stable sac. For 145 patients, their AAA remodeling category was determined by US-US comparison (shrinkage: 55 (82%), stable: 90 (84%)). The AAA remodeling category of the other 29 patients was determined by CT-CT comparison (shrinkage: 12 (18%), stable: 17 (16%)).

Baseline demographics and clinical characteristics were computed for the total population, and separately for the shrinkage and stable group—as shown in Table 1. For the included patients, the mean age at the time of the procedure was 72 years (range 53–92 years), and 86% were male. Medtronic Endurant (n = 102, 59%) was the most frequently used endograft, followed by Gore Excluder (n = 51, 29%), Endologix AFX (n = 16, 9%), Cook Zenith (n = 3, 2%), and Vascutek Anaconda (n = 2, 1%).

No significant differences were found between the two groups in terms of demographics, risk scores, atherosclerotic risk factors, comorbidities, and medication usage. The shrinkage group did show a significantly higher hemoglobin level—even though the absolute difference was only 0.2 mmol/L (9.1 ± 0.8 vs. 8.9 ± 0.9, *p* = 0.045).

Preoperative AAA- and EVAR-related characteristics were also computed for the total population, and separately for the shrinkage and stable group—as shown in Table 2. No significant differences were found in terms of preoperative AAA geometry, device type, procedural characteristics, residual endoleaks, or hospitalization.

To identify predictors of AAA sac shrinkage, baseline characteristics were analyzed with univariate and multivariable logistic regression analysis—for which the main results are shown in Table 3 (all results in Table S1). On univariate analysis, no significant predictors were found.

Thirteen variables had a *p*-value < 0.3 and were considered for inclusion in the multivariable logistic regression model, including age, gender, ASA classification, hypertension, inflammatory diseases, pulmonary history, hemoglobin level, GFR, antiplatelet therapy, statins, infrarenal β angle, maximum AAA diameter, and days at the hospital. Only six of these variables could be included in the multivariable logistic regression analysis, as the number of events was 67. Age, hemoglobin level, and GFR had *p* < 0.1 on univariate analysis and were thus included in the manual stepwise logistic regression analysis. In addition, gender, maximum AAA diameter, and infrarenal β angle were included based on their *p*-values and expected predictive value. Based on the manual stepwise logistic regression analysis, age, infrarenal β angle, and maximum AAA diameter were entered in the multivariable logistic regression analysis to ascertain their effects on the likelihood that patients develop a shrinking AAA.

The multivariable logistic regression model indicated that larger preoperative maximum AAA diameter was associated with an increased likelihood of developing AAA shrinkage with an OR of 1.05 (95% CI 1.004–1.09, *p* = 0.031), and older age at the time of procedure and larger preoperative infrarenal β angle were associated with a reduction in the likelihood of developing AAA shrinkage with respective ORs of 0.95 (95% CI 0.91–0.995, *p* = 0.027) and 0.98 (95% CI 0.91–0.995, *p* = 0.045). This regression model was statistically significant, χ^2^(3) = 11.382, *p* = 0.010. It explained 8.7% (Nagelkerke R^2^) of the variance in AAA remodeling and correctly classified 66.7% of the patients with AAA shrinkage from the total population.

The relation between the preoperative maximum AAA diameter and development of AAA shrinkage was further evaluated by assessing the Pearson’s correlation coefficient of the preoperative maximum AAA diameter and the absolute change in AAA diameter within the first year after EVAR. The positive correlation demonstrated that in patients with a preoperative large AAA diameter, the diameter changed more than in patients with a preoperative small diameter (r = 0.174, *p* = 0.022).

**Figure 2:**
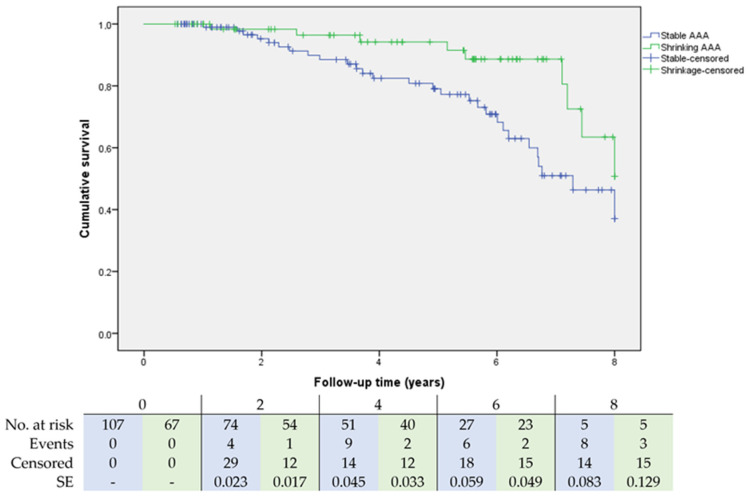
Kaplan-Meier survival analysis of freedom from death for stable and shrinking AAA through eight years. Number at risk represents patients at risk at that timepoint; events and censored were evaluated at end of interval. A blue color represents the stable AAA group, and a green color represents the shrinking AAA group. SE = standard error.

A total of 37 (21.3%) patients died after a mean of 4.7 ± 2.2 years after EVAR. In the shrinkage group, nine (16.7%) patients were deceased after a mean of 5.4 ± 2.5 years after EVAR, and in the stable group 28 (32.6%) patients were deceased after a mean of 4.5 ± 2.1 years (percentage: *p* = 0.038, time: *p* = 0.309). Figure 2 shows the Kaplan-Meier survival curves for shrinking and stable AAA. The log-rank test demonstrated that the survival distributions significantly differed between shrinking and stable AAA (*p* = 0.019). One EVAR-related death occurred in the stable group after 2.5 years due to an infected endograft—treated with antibiotics and open surgical removal of the endograft with insertion of a tube graft. However, postoperatively, the patient developed pneumonia and infection of the new AAA graft, which was complicated by renal insufficiency, ultimately leading to the patient’s death. No EVAR-related death occurred in the shrinkage group. Other causes of death in the stable vs. shrinkage group were: neoplasm (8 vs. 1), cardiac (3 vs. 1), pulmonary (2 vs. 0), focusless infection (1 vs. 0), perforated diverticulitis (1 vs. 0), renal (0 vs. 1), and unknown (12 vs. 6).

## 4. Discussion

This research showed a significantly lower all-cause mortality in patients with a shrinking AAA one-year after EVAR, compared to patients with a stable AAA, confirming data of previous studies [7,8,9]. In addition, one death of a patient with a stable AAA was EVAR-related, while none of the patients with AAA shrinkage died from this cause. A larger preoperative maximum AAA diameter is associated with an increased likelihood of developing AAA shrinkage one-year after EVAR—whereas older age and a larger preoperative infrarenal β angle are associated with a reduced likelihood of AAA shrinkage. It should be noted, however, that the observed odds ratios demonstrated minor associations, and neither of these variables could independently predict AAA shrinkage on the univariate analysis. The multivariable logistic model could correctly predict two-thirds of the shrinking AAAs out of the total cohort. This indicates that the found predictors cannot be used yet in clinical care, but they do warrant attention for further research in order to be able to predict aneurysm remodeling after EVAR.

The evidence on a relationship between the preoperative AAA diameter and remodeling after EVAR varies greatly between different published studies—as shown in a recent review [13]. When focusing on studies reporting specifically on AAA remodeling one-year after EVAR, two studies found no correlation, while two others observed that a larger preoperative AAA diameter was related to an increased likelihood of AAA shrinkage [22,23,24,25]—consistent with the findings of this analysis. However, this observation might be distorted by the focus of this study, namely comparing shrinking with stable AAA after EVAR, consequently excluding growing AAAs. If the calculation of the Pearson’s coefficient between the preoperative AAA diameter and the absolute diameter change would be performed in a group with shrinking, stable, and growing AAAs (n = 192), it would demonstrate that a preoperative larger diameter is related to more AAA remodeling in general—so more shrinkage as well as growth (r = 0.150, *p* = 0.038).

Older age was found to be associated with a reduction in the likelihood of developing AAA shrinkage—similar to several other reports [26,27,28,29]. This relation might be attributed to lower elastic properties of the AAA wall in the elderly, which may contribute to the process of aortic remodeling after EVAR [30].

Another negative predictive measure for AAA shrinkage was a larger preoperative infrarenal β angle. A recent systematic review demonstrated that two other studies that investigated AAA remodeling one-year after EVAR did not find significant relations between infrarenal angulation and AAA remodeling—in contrast to this study [13]. However, both studies were not comparable to this study as one study focused only on the Lombard Aorfix high angulation device and used another definition for neck angle [25], while the other study demonstrated smaller infrarenal angles with an undescribed measurement method of these angles [24].

When comparing this study to others, the percentages of patients presenting with a growing, stable, and shrinking AAA one-year after EVAR were comparable [11,31]. Furthermore, for the mean age, preoperative maximum AAA diameter, and incidence of type II endoleak, this cohort was comparable to other studies [12]. In addition, this sample size was similar to other single-center studies for an inclusion period of eight years [12].

This study also indicates some recommendations for future research to obtain more robust evidence on predictors of AAA shrinkage and its corresponding better long-term outcomes. This could also apply for other aortic segments, including the abdominal and thoracic aorta. As the findings on the predictive value of the preoperative AAA diameter are so variable, we recommend investigation of the influence of the intraluminal thrombus, luminal volume, and total AAA volume on AAA shrinkage after EVAR, as the maximum AAA diameter is composed of these volumes and they might be more sensitive in predicting AAA remodeling. Oliveira-Pinto et al. already showed that a larger AAA luminal volume is a risk factor for late complications after EVAR [32]. In addition, artificial intelligence and radiomics could be applied to improve the identification of predictors of AAA shrinkage [33,34]. Ding et al. already showed that early postoperative CT texture analysis is a better predictor of AAA growth than clinical factors and conventional imaging evaluation together [35]. In general, we emphasize the use of standardized measurement of the maximum AAA diameter before and after EVAR and encourage future studies to report the presence or absence of AAA shrinkage of ≥5 mm one-year after EVAR [7,8,9,10,11].

If AAA remodeling after EVAR, and the corresponding long-term outcomes, could be reliably predicted, treatment choices could be made to increase the chances of a good outcome. For instance, the decision between EVAR and open surgery could be reconsidered. Furthermore, the EVAR procedure might be optimized in selected patients with a lower likelihood of AAA shrinkage by including active sac management—such as embolization of side branches or filling the aneurysmal sac—prior to the procedure [36,37]. Reliable prediction of AAA remodeling could also aid stratification of the follow-up surveillance after EVAR based on the patient’s individual risk. Currently, most surveillance programs are uniform for all patients—irrespective of the presence or absence of predictive indicators [38]. By developing risk-based surveillance programs, the burden of follow-up could be drastically reduced since 40–50% of the patients experience AAA shrinkage one-year after EVAR [7,31,39]. This would not only decrease surveillance-related costs, but also the strain and psychological burden of hospital visits on patients.

This research has several limitations. Since this is a retrospective study, it was subjected to selection and information biases. Furthermore, as a single-center study, overall generalizability of the results might be limited. To prevent confounding of predictors of AAA sac remodeling, inclusion and exclusion criteria were set more extensively than in comparable studies. Patients with non-elective procedures or AAA-related reinterventions within the first year were excluded, as well as patients without primary assisted technical success or without imaging with similar modalities pre-, and postoperatively. It is of utmost importance that, when assessing the diameter, the same imaging modality is used at each time point as this may cause an inclusion bias. A drawback of this method was that 173 patients had to be excluded because of these strict criteria. Furthermore, bias was introduced by focusing only on stable vs. shrinking AAA, while leaving out growing AAA. As a result, relations between a variable and more/less AAA remodeling might be incorrectly depicted in this study as a relation between this variable and more/less AAA shrinkage.

In conclusion, patients with a shrinking AAA, one-year after EVAR, have better long-term survival compared to patients with a stable AAA size. Preoperative age, maximum AAA diameter, and infrarenal β angle showed to be useful in preoperatively differentiating patients that will have a shrinking AAA at one-year follow up from those with a stable AAA diameter. The final prediction model could correctly predict AAA shrinkage in two-thirds of the total patients—but this is not sufficient enough to be used in clinical practice yet. Future research should therefore dive deeper into the influence of AAA anatomy on AAA shrinkage after EVAR and explore potential predictors with artificial intelligence and radiomics.

## Figures and Tables

**Table 1:** Baseline demographics and clinical characteristics of the study population stratified by AAA shrinkage.

Variables	Total Population	Stable	Shrinkage	*p*-Value
Number of patients	174	107 (61.5)	67 (38.5)	
Age (years)	71.8 ± 7.6	72.6 ± 7.3	70.4 ± 7.9	0.061
Male sex	149 (85.6)	88 (82.2)	61 (91.0)	0.107
BMI ^1^ (kg/m^2^)	26.7 ± 3.7	26.9 ± 4.0	26.4 ± 3.1	0.361
Systolic blood pressure (mmHg)	142 ± 21	142 ± 23	142 ± 20	0.969
Diastolic blood pressure (mmHg)	80 ± 10	80 ± 11	81 ± 10	0.609
ASA ^2^ classification				0.734
1	2 (1.2)	1 (0.9)	1 (1.5)	
2	94 (54.3)	55 (51.4)	39 (59.1)	
3	70 (40.5)	46 (43.0)	24 (36.4)	
4	7 (4)	5 (4.7)	2 (3.0)	
SVS/AAVS ^3^ risk score	7.5 ± 5.2	7.8 ± 5.2	7.0 ± 5.2	0.383
SVS/AAVS risk score 0–3	0.77 ± 0.67	0.81 ± 0.68	0.71 ± 0.65	0.350
SVS/AAVS risk score category				0.628
Absent	57 (36.1)	34 (34.0)	23 (39.7)	
Mild	80 (50.6)	51 (51.0)	29 (50.0)	
Moderate	21 (13.3)	15 (15.0)	6 (10.3)	
SGVI ^4^ score	3.1 ± 0.5	3.1 ± 0.5	3.0 ± 0.5	0.735
SGVI score high risk	11 (7.7)	7 (7.9)	4 (7.4)	0.921
Risk factors				
Smoking	60 (36.1)	34 (33.7)	26 (40.0)	0.407
Diabetes mellitus	31 (17.8)	17 (15.9)	14 (20.9)	0.401
Hypertension	123 (70.7)	79 (73.8)	44 (65.7)	0.250
Hyperlipidemia	134 (84.3)	81 (82.7)	53 (86.9)	0.476
Inflammatory diseases	27 (15.7)	19 (18.1)	8 (11.9)	0.279
Comorbidities				
Cardiac status	77 (46.7)	50 (48.5)	27 (43.5)	0.533
Renal status	48 (27.9)	31 (29.2)	17 (25.8)	0.620
Pulmonary status	37 (22.0)	26 (25.0)	11 (17.2)	0.235
Coronary artery disease	15 (8.6)	10 (9.3)	5 (7.5)	0.667
COPD ^5^	29 (16.7)	20 (18.7)	9 (13.4)	0.365
Lab results				
Hemoglobin (mmol/L)	9.0 ± 0.9	8.9 ± 0.9	9.1 ± 0.8	0.045
Leukocytes (×10^9^/L)	8.4 ± 2.5	8.5 ± 2.7	8.2 ± 2.0	0.441
Creatinine (µmol/L)	93 ± 30	94 ± 32	91 ± 28	0.428
GFR ^6^ (mL/min/1.73 m^2^)	70 ± 17	69 ± 18	73 ± 16	0.094
Medication				
Anticoagulant therapy	141 (84.9)	87 (85.3)	54 (84.4)	0.872
Antiplatelet therapy	146 (83.9)	86 (80.4)	60 (89.6)	0.109
Metformin	26 (14.9)	15 (14.0)	11 (16.4)	0.666
Statins	135 (77.6)	80 (74.8)	55 (82.1)	0.260

Continuous data are presented as mean ± standard deviation, categorical data are presented as number (%). ^1^ BMI = body mass index, ^2^ ASA = American Society of Anesthesiologists, ^3^ SVS/AAVS = Society for Vascular Surgery/American Association for Vascular Surgery, ^4^ SGVI = St George’s Vascular Institute, ^5^ COPD = chronic obstructive pulmonary disease, ^6^ GFR = glomerular filtration rate.

**Table 2:** Baseline AAA- and EVAR-related characteristics of the study population stratified by AAA shrinkage.

Variables	Total Population	Stable	Shrinkage	*p*-Value
Number of patients	174	107 (61.5)	67 (38.5)	
Preoperative AAA ^1^ geometry				
Infrarenal neck diameter (mm)	23.7 ± 3.2	23.9 ± 3.4	23.4 ± 3.0	0.395
Infrarenal neck length (mm)	29.4 ± 13.2	30.0 ± 13.6	28.3 ± 12.7	0.421
Infrarenal β angle (°)	51.7 ± 16.3	53.2 ± 16.8	49.2 ± 15.3	0.121
Maximum AAA diameter (mm)	54.3 ± 8.6	53.7 ± 9.1	55.3 ± 7.6	0.221
Maximum CIA diameter (mm)	18.6 ± 8.0	19.1 ± 7.9	17.9 ± 8.2	0.392
Maximum EIA ^2^ diameter (mm)	9.4 ± 2.5	9.3 ± 2.0	9.6 ± 3.2	0.449
Device				0.982
Medtronic Endurant	102 (58.6)	64 (59.8)	38 (56.7)	
Gore Excluder	51 (29.3)	31 (29.0)	20 (29.9)	
Endologix AFX	16 (9.2)	9 (8.4)	7 (10.4)	
Cook Zenith	3 (1.7)	2 (1.9)	1 (1.5)	
Vascutek Anaconda	2 (1.1)	1 (0.9)	1 (1.5)	
Graft material				0.701
Polyester	107 (61.5)	67 (62.6)	40 (59.7)	
PTFE ^3^	67 (38.5)	40 (37.4)	27 (40.3)	
Blood loss (mL)	183 ± 313	177 ± 293	193 ± 346	0.746
Procedure time (min)	98 ± 45	100 ± 46	95 ± 42	0.459
Perioperative residual endoleak	36 (20.9)	22 (21.0)	14 (20.9)	0.993
Type I endoleak	0 (0)	0 (0)	0 (0)	NA ^4^
Type II endoleak	33 (19.0)	19 (17.8)	14 (20.9)	0.607
Type III endoleak	0 (0)	0 (0)	0 (0)	NA
Type IV endoleak	1 (0.6)	1 (0.9)	1 (0.9)	0.427
Days at hospital	3.4 ± 1.9	3.6 ± 2.0	3.2 ± 1.7	0.139
Days at ICU ^5^	0.01 ± 0.08	0.01 ± 0.1	0.00 ± 0.00	0.417
Complications during hospitalization	31 (17.9)	20 (18.9)	11 (16.4)	0.682

*p*-value for difference between stable and shrinkage group. Continuous data are presented as mean ± standard deviation, categorical data are presented as number (%). ^1^ AAA = abdominal aortic aneurysm, ^2^ EIA = external iliac artery, ^3^ PTFE = polytetrafluoretheen, ^4^ NA = not applicable, ^5^ ICU = intensive care unit.

**Table 3:** Logistic regression analysis of baseline characteristics for AAA sac shrinkage.

	Univariate Analysis		Multivariable Analysis	
	OR (95% CI)	*p*-Value	OR (95% CI)	*p*-Value
Age (years)	0.96 (0.92–1.00)	0.063	0.95 (0.91–0.995)	0.027
Male sex	2.20 (0.83–5.82)	0.114	NA	
Hemoglobin (mmol/L)	1.47 (1.00–2.16)	0.051	NA	
GFR (mL/min/1.73 m^2^)	1.02 (1.00–1.04)	0.096	NA	
Infrarenal β angle (°)	0.99 (0.97–1.00)	0.122	0.98 (0.91–0.995)	0.045
Maximum AAA diameter (mm)	1.02 (0.99–1.06)	0.223	1.05 (1.004–1.09)	0.031

## Data Availability

The data presented in this study are available on request from the corresponding author. The data are not publicly available due to privacy reasons.

## Supplementary Materials

The following are available online at https://www.mdpi.com/article/10.3390/jcm11051394/s1, Supplementary Table S1: Logistic regression analysis of all baseline characteristics for AAA sac shrinkage.
