# Supplementary material for: Predictors of Abdominal Aortic Aneurysm Shrinkage after Endovascular Repair
Source: J Clin Med. 2022 Mar 3;11(5):1394. doi: 10.3390/jcm11051394 (PMC8910935; doi:10.3390/jcm11051394)
Supplement: Supplementary file 1 [file jcm-11-01394-s001.zip › jcm-1593045-supplementary.pdf]

Journal of
Clinical Medicine

Supplementary Table S1. Logistic regression analysis of all baseline characteristics for abdominal aortic aneurysm (AAA) sac shrinkage.

	Univariate Analysis		Multivariable Analysis	
	**OR (95% CI)**	***p* Value**	**OR (95% CI)**	***p* Value**
Age (years)	0.96 (0.92–1.00)	0.063	0.95 (0.91–0.995)	0.027
Male sex	2.20 (0.83–5.82)	0.114	NA [1]	
BMI [2] (kg/m^2)	0.96 (0.89–1.05)	0.384	NA	
Systolic blood pressure (mmHg)	1.00 (0.99–1.01)	0.969	NA	
Diastolic blood pressure (mmHg)	1.01 (0.98–1.04)	0.607	NA	
ASA [3] classification, continuous	0.74 (0.44–1.25)	0.261	NA	
SVS/AAVS [4] risk score, continuous	0.97 (0.91–1.04)	0.381	NA	
SVS/AAVS risk score 0-3, continuous	0.79 (0.48–1.29)	0.348	NA	
SGVI [5] score	0.89 (0.45–1.76)	0.733	NA	
SGVI score high risk, continuous	0.94 (0.26–3.36)	0.921	NA	
SGVI score high risk, categorial	1.07 (0.30–3.83)	0.921	NA	
Risk factors				
Smoking	0.76 (0.40–1.45)	0.407	NA	
Diabetes mellitus	1.40 (0.64–3.07)	0.402	NA	
Hypertension	0.68 (0.35–1.32)	0.251	NA	
Hyperlipidemia	1.39 (0.56–3.45)	0.477	NA	
Inflammatory diseases in history	1.63 (0.67–3.97)	0.282	NA	
Comorbidities				
Cardiac status	0.82 (0.43–1.52)	0.534	NA	
Renal status	0.84 (0.42–1.68)	0.620	NA	
Pulmonary status	0.62 (0.28–1.37)	0.238	NA	
Coronary artery disease	1.28 (0.42–3.92)	0.667	NA	
COPD [6]	1.48 (0.63–3.48)	0.367	NA	
Lab results				
Hemoglobin (mmol/L)	1.47 (1.00–2.16)	0.051	NA	
Leukocytes (x10^9/L)	0.95 (0.82–1.10)	0.474	NA	
Creatinine (µmol/L)	1.00 (0.99–1.01)	0.427	NA	
GFR [7] (mL/min/1.73m^2)	1.02 (1.00–1.04)	0.096	NA	
Medication				
Anticoagulant therapy	1.07 (0.45–2.56)	0.872	NA	
Antiplatelet therapy	0.48 (0.19–1.20)	0.114	NA	
Metformin	0.83 (0.36–1.93)	0.666	NA	
Statins	0.65 (0.30–1.39)	0.262	NA	
Preoperative AAA geometry				
Infrarenal neck diameter (mm)	0.95 (0.86–1.06)	0.393	NA	
Infrarenal neck length (mm)	0.99 (0.97–1.02)	0.419	NA	
Infrarenal β angle (°)	0.99 (0.97–1.00)	0.122	0.98 (0.91–0.995)	0.045
Maximum AAA diameter (mm)	1.02 (0.99–1.06)	0.223	1.05 (1.004–1.09)	0.031
Maximum CIA [8] diameter (mm)	0.98 (0.94–1.03)	0.392	NA	
Maximum EIA [9] diameter (mm)	1.06 (0.91–1.23)	0.452	NA	
Device type		0.982	NA	
Graft material	1.13 (0.61–2.11)	0.701	NA	
Blood loss (mL)	1.00 (0.999–1.001)	0.745	NA	
Procedure time (min)	1.00 (0.99–1.01)	0.460	NA	
Perioperative residual endoleak	0.99 (0.47–2.12)	0.993	NA	
Days at hospital	0.88 (0.73–1.05)	0.144	NA	
Complications during hospitalization	1.18 (0.53–2.66)	0.683	NA	

[1] NA = not applicable, [2] BMI = body mass index, [3] ASA = American Society of Anesthesiologists, [4] SVS/AAVS = Society for Vascular Surgery/American Association for Vascular Surgery, [5] SGVI = St George's Vascular Institute, [6] COPD = chronic obstructive pulmonary disease, [7] GFR = glomerular filtration rate, [8] CIA = common iliac artery, [9] EIA = external iliac artery.
